# Vitamin D in pediatric patients with obesity and arterial hypertension

**DOI:** 10.1038/s41598-021-98993-8

**Published:** 2021-10-01

**Authors:** Živa Radulović, Zarja Polak Zupan, Aljoša Tomazini, Nataša Marčun Varda

**Affiliations:** 1 Medical Faculty, University of Maribor, Taborska ulica 8, 2000 Maribor, Slovenia; 2 Department of Paediatrics, Paediatric Nephrology and Hypertension Unit, University Medical Centre Maribor, Ljubljanska ulica 5, 2000 Maribor, Slovenia

**Keywords:** Health care, Medical research, Nephrology

## Abstract

The purpose of this study was to find potential differences in vitamin D levels between different groups: overweight children with hypertension, normal-weight children with hypertension, overweight children with normal blood pressure and normal-weight children without hypertension, representing the control group. We also wanted to determine whether there are correlations between vitamin D levels and other clinical laboratory parameters, to evaluate the potential need for substitution. We measured vitamin D, homocysteine, total cholesterol, HDL, LDL, triglycerides, uric acid, glucose, apolipoprotein A1, apolipoprotein B, alkaline phosphatase, calcium, phosphate and magnesium serum levels in all groups. We also took anthropometric measurements (body weight, height, body mass index (BMI)) and observed patients’ blood pressure. The results were analyzed with SPSS statistic tool with basic statistical methods. The study included 175 children between 5 and 18 years of age. Fiftyseven were healthy (group A—control group), 41 normal-weight with hypertension (group B), 44 overweight with hypertension (group C) and 33 overweight with normal blood pressure (group D). The results showed statistically significant differences in values of vitamin D between all groups—A and B (p = 0.003), A and C (p < 0.001), A and D (p < 0.001), B and D (p = 0.043), B and C (0.030), except for groups C and D (p = 0.830). There were statistically significant correlations between vitamin D and BMI (r = − 0.196, p = 0.010), systolic pressure (r = − 0.190, p = 0.002), diastolic pressure (r = − 0.149, p = 0.050), homocysteine (r = − 0.208, p = 0.007), triglycerides (r = − 0.196, p = 0.011) and apolipoprotein A1 (r = 0.222, p = 0.007), confirmed in multivariate model. For the blood pressure, the higher the systolic blood pressure, the lower the average vitamin D was. The pilot study shows significant differences in serum vitamin D levels between all groups of children, apart from groups C and D. These results, combined with statistically significant correlations between vitamin D and systolic and diastolic blood pressure suggest the need for monitoring and potential substitution of vitamin D in in pediatric patients with hypertension.

## Introduction

Vitamin D is one of the most vital elements in the human body as it interferes with many physiological and, in some cases, pathological processes. Its role in bone metabolism has long been recognized, however, newer studies have also revealed its importance in many other processes, such as autoimmunity, inflammation, angiogenesis as well as arterial hypertension^1,2^. There is also recent evidence of correlations between vitamin D and other important parameters and determinants of health in adults and children^3,4^.

The prevalence of childhood obesity in developed countries is still increasing, and the same goes for obesity-related health problems^5^. The studies so far have shown vitamin D deficiency in overweight children, which is suggested to be the consequence of volume dilution, reduced sunlight exposure as well as the accumulation of vitamin D in the fat tissue thus reducing its bioavailability, which can further accelerate pathological processes. Because of large differences in weight between children of different sex and ages, we used body mass index percentiles for the purpose of our study, so we could compare measurements more accurately. The causes of obesity can be mainly attributed to lifestyle factors, which include over-eating and under-exercising, but they can also have genetic and hormonal basis^6,7^.

Arterial hypertension is also not uncommon in children. It affects 1 – 4% of children, either as primary hypertension, which results from unhealthy lifestyle and genetic predispositions, or as secondary hypertension, which can be caused by different, most often nephrological diseases. The former is more common in older children. On the other hand, different diseases can affect children of any age; therefore, secondary hypertension can occur at any time^8^. Some studies have concluded that children with lower vitamin D levels have significantly higher blood pressure. This is the consequence of the effect vitamin D has on lowering the activity of the renin–angiotensin hormone system. It also affects many other parameters and risk factors such as blood lipid parameters through its actions on the vitamin D receptor (VDR)^9,10^.

As a result, we can see how recognizing the key role of vitamin D in various pathological processes may help us to understand and potentially treat different pathologies, some of which can start in early childhood and affect health much sooner than previously expected^10^.

The purpose of this study was to find potential differences in vitamin D serum levels between three groups of patients: overweight children with normal blood pressure, normal-weight children with hypertension and overweight children with hypertension. We also wanted to determine whether there are any correlations between vitamin D and other clinical as well as laboratory parameters, to evaluate potential need for its substitution.

## Materials and methods

The study included 175 children and adolescents between 5 and 18 years of age. Fifty-seven were healthy, 41 had hypertension, 44 were overweight with hypertension and 33 were just overweight. The measurements were obtained between November 2019 and November 2020 under the supervision of the Department of Pediatrics, University Medical Centre Maribor. The study was approved by Ethical Committee of Maribor University Medical Centre in November 2019. All methods were carried out in accordance with relevant guidelines and regulations. All participants or their legal guardians signed an informed consent.

Patients were recruited for the study during hospitalization that was indicated for cardiovascular assessment. Anthropometric measurements (body weight and height) were taken and every patient's BMI calculated. We measured blood pressure under the Second Task Force recommendations, which we also used in diagnosing our patients^11^. We also conducted ambulatory 24-h blood pressure monitoring^8^. The BMI scores were used to determine the accurate percentiles for patients' sex and age^12,13^.

Required sample size was calculated and determined to be at least 173 to have a confidence level of 95% for the real value to be within ± 5%^14^. BMI score above 95th percentile for age and gender was used to define the overweight population^13^. Hypertension was diagnosed as systolic or diastolic blood pressure ≥ 95th percentile for the sex, age, and height, measured at three different clinical situations. Hypertension was also confirmed with 24-h blood pressure monitoring^11^. All children and adolescents with chronic diseases or comorbidities (except hypertension and/or obesity) that could affect the measured parameters were excluded.

The blood samples were obtained from children in the fasted state and were used to determine specific laboratory parameters under standard procedures: homocysteine, total cholesterol, LDL cholesterol, HDL cholesterol, triglycerides (TG), uric acid, glucose, apolipoprotein A1 (apo A), apolipoprotein B (apo B), alkaline phosphatase, calcium, phosphate, and magnesium^15^. Serum vitamin D levels were determined in nmol/liter using immunochemical, a protein binding method (Cobas Roche)^15,16^.

The results were statistically analyzed with the help of SPSS Statistics software (IBM, version 25). Normality of the data was assessed using steam-and leaf plot, skewness and kurtosis. Upon the results, the normal distribution of the data was found. For analysis, basic statistical methods were used e.g. for descriptive statistics (the mean values with the standard deviations), the Analysis of variance (ANOVA) tests and bivariate correlations. Strength of correlation was defined by the Pearson coefficient (r), where a weak correlation ranged between 0 and 0.3, a moderate one with r between 0.3 and 0.7, and a strong correlation with r between 0.7 and 1. Multivariate regression analysis was also performed. A significant statistical difference was defined as p < 0.05.

## Results

### Sample characteristics and epidemiology

The study included 175 children and adolescents, 76 girls (43.3%) and 99 boys (56.7%), between 5 and 18 years of age. Among these, 57 (32.6%) were healthy children that represented our control group (group A). Group B was comprised of 41 (23.4%) children with hypertension, group C included 44 (25.1%) overweight children with hypertension and group D was represented by 33 (18.9%) overweight children with normal blood pressure. The measurements were obtained between November 2019 and November 2020. The mean age of the subjects was 13.8 ± 3.54 years.

Table 1 shows mean values of clinical and anthropometric parameters of all children included in the study.

**Table 1:** Descriptive statistics with mean values and standard deviations of clinical and laboratory parameters of all included subjects.

Variable	Mean	Standard deviation
Age [years]	13.8	± 3.54
Vitamin D [nmol/L]	63.44	± 24.39
BMI percentile	76.17	± 27.47
Systolic pressure [mmHg]	122	± 10.45
Diastolic pressure [mmHg]	68	± 8.39
Homocysteine [μmol/L]	8.15	± 2.44
Total cholesterol [mmol/L]	4.50	± 0.81
LDL cholesterol [mmol/L]	2.78	± 0.72
HDL cholesterol [mmol/L]	1.42	± 0.36
TG [mmol/L]	1.07	± 0.55
Uric acid [μmol/L]	303.41	± 79.07
Glucose [mmol/L]	4.74	± 0.52
ApoA [g/L]	1.45	± 0.42
ApoB [g/L]	0.76	± 0.20
Alkaline phosphatase [µkat/L]	3.28	± 1.80
Calcium [mmol/L]	2.39	± 0.12
Phosphate [mmol/L]	1.38	± 0.64
Magnesium [mmol/L]	0.90	± 0.09

*BMI Percentile* body mass index percentile, *LDL cholesterol* low-density lipoprotein cholesterol, *HDL cholesterol* high-density lipoprotein cholesterol, *TG* triglycerides, *ApoA* apolipoprotein A1, *ApoB* apolipoprotein B.

In Table 2 we can see comparisons of different variables between the groups studied and the control group.

**Table 2:** Descriptive statistic and comparison of parameters between the groups (independent samples t-test).

Variable	Control group	Patients with hypertension		Overweight patients with hypertension		Overweight patients with normal blood pressure	
	Mean (min, max)	Mean (min, max)	p-value (A and B)	Mean (min, max)	p-value (A and C)	Mean (min, max)	p-value (A and D)
Age [years]	13.0 (6, 18)	15.1 (8, 18)	0.002**	13.4 (5, 18)	0.672	13.8 (7, 18)	0.299
Vitamin D [nmol/L]	78.59 (35.9, 148.5)	62.7 (7.5, 113.2)	0.003**	52.17 (13.5, 90.9)	p < 0.001**	53.23 (7.5, 83.9)	p < 0.001**
BMI percentile	56 (2, 95)	64.97 (5.0, 94.0)	0.114	97.56 (91.0, 99.0)	p < 0.001**	95.69 (86.0, 99.0)	p < 0.001**
Systolic pressure [mmHg]	113 (97, 130 )	133 (124, 148)	p < 0.001**	126 (110, 140)	p < 0.001**	117 (103, 135)	0.079
Diastolic pressure [mmHg]	65 (54, 94)	75 (61, 93)	p < 0.001**	69 (55, 77)	p < 0.001**	67 (47, 79)	0.732
Homocysteine [μmol/L]	6.75 (1.8, 15.2)	7.63 (3.0, 18.2)	0.139	9.38 (5.7, 9.9)	p < 0.001**	9.46 (7.6, 11.0)	p < 0.001**
Total cholesterol [mmol/L]	4.53 (2.9, 6.95)	4.21 (2.78, 6.10)	0.051	4.66 (2.29, 6.94)	0.472	4.57 (3.35, 6.33)	0.830
LDL cholesterol [mmol/L]	2.73 (1.38, 4.9)	2.48 (1.5, 4.0)	0.091	3.01 (0.9, 5.10)	0.074	2.92 (1.90, 4.50)	0.211
HDL cholesterol [mmol/L]	1.57 (0.76, 2.25)	1.46 (0.89, 2.53)	0.130	1.32 (0.8, 2.66)	0.002**	1.29 (0.81, 2.23)	0.001**
TG [mmol/L]	0.87 (0.34, 1.67)	1.03 (0.45, 2.61)	0.089	1.29 (0.30, 4.18)	0.001**	1.11 (0.57, 3.31)	0.022*
Uric acid [μmol/L]	262.0 (121.0, 424.0)	310.0 (101.0, 436.0)	0.006**	336.75 (187.0, 534.0)	p < 0.001**	322.0 (166.0, 477.0)	0.001**
Glucose [mmol/L]	4.76 (1.42, 6.4)	4.71 (4.1, 5.6)	0.628	4.71 (3.6, 5.9)	0.666	4.77 (3.6, 5.4)	0.957
ApoA [g/L]	1.64 (1.04, 6.87)	1.42 (0.99, 1.94)	0.101	1.37 (1.07, 1.77)	0.029*	1.36 (1.06, 1.62)	0.036*
ApoB [g/L]	0.69 (0.4, 1.10)	0.70 (0.47, 1.1)	0.678	0.82 (0.29, 1.33)	0.003**	0.83 (0.40, 1.58)	0.009**
Alkaline phosphatase [µkat/L]	3.33 (1.04, 6.87)	3.34 (0.91, 9.64)	0.995	3.17 (0.98, 6.24)	0.649	3.26 (1.36, 6.83)	0.858
Calcium [mmol/L]	2.39 (2.23, 2.75)	2.42 (2.22, 2.64)	0.110	2.39 (2.20, 2.52)	0.741	2.34 (1.57, 2.54)	0.173
Phosphate [mmol/L]	1.38 (0.85, 1.85)	1.28 (0.74, 1.80)	0.043*	1.34 (0.75, 1.9)	0.472	1.33 (0.77, 2.44)	0.471
Magnesium [mmol/L]	0.9 (0.73, 1.28)	0.89 (0.67, 1.05)	0.673	0.92 (0.71, 1.07)	0.228	0.89 (0.32, 1.32)	0.648

Control group (A), children with hypertension (B), overweight children with hypertension (C) and overweight children with normal blood pressure (D). Value p represents the comparison between the control group and all the other groups studied.

*Min* minimal, *max* maximal, *BMI Percentile* body mass index percentile, *LDL cholesterol* low-density lipoprotein cholesterol, *HDL cholesterol* high-density lipoprotein cholesterol, *TG* triglycerides, *ApoA* apolipoprotein A1, *ApoB* apolipoprotein B.

*p < 0.05, **p < 0.01.

The results show differences in variables between the groups studied and the control group. Statistically significant differences in vitamin D levels were found in all the groups. In group with hypertension we found statistically significant differences in systolic pressure, diastolic pressure, uric acid and phosphate compared to the control group. Overweight patients with hypertension had important statistical differences in BMI percentiles, systolic and diastolic pressure, homocysteine, HDL cholesterol, TG, uric acid, ApoA and ApoB compared to the control group. Adipose children with normal arterial pressure and control group statistically differed in BMI, homocysteine, HDL cholesterol, TG, uric acid, ApoA and ApoB.

Table 3 exhibits data analyzed with independent samples t-test between the groups studied for all the variables.

**Table 3:** Comparison of the variables with the independent samples t-test between all the groups.

Variable	C and D	B and D	B and C	Total p—value
Vitamin D [nmol/L]	p = 0.830	p = 0.043*	p = 0.030*	p < 0.001**
BMI percentile	p = 0.013*	p < 0.001**	p < 0.001**	p < 0.001**
Systolic pressure [mmHg]	p < 0.001**	p < 0.001**	p < 0.001**	p < 0.001**
Diastolic pressure [mmHg]	p = 0.026*	p < 0.001**	p = 0.001**	p < 0.001**
Homocysteine [μmol/L]	p = 0.658	p < 0.001**	p = 0.001**	p < 0.001**
Total cholesterol [mmol/L]	p = 0.637	p = 0.045*	p = 0.015*	p = 0.314
LDL cholesterol [mmol/L]	p = 0.552	p = 0.004**	p = 0.001**	p = 0.019*
HDL cholesterol [mmol/L]	p = 0.727	p = 0.040*	p = 0.099	p = 0.001**
TG [mmol/L]	p = 0.184	p = 0.503	p = 0.055	p = 0.001**
Glucose [mmol/L]	p = 0.572	p = 0.517	p = 0.975	p = 0.639
ApoA [g/L]	p = 0.777	p = 0.132	p = 0.232	p = 0.075
ApoB [g/L]	p = 0.862	p = 0.019*	p = 0.008**	p = 0.003**
Alkaline phosphatase [µkat/L]	p = 0.836	p = 0.888	p = 0.742	p = 0.487
Uric acid [μmol/L]	p = 0.461	p = 0.545	p = 0.154	p < 0.001**
Calcium [mmol/L]	p = 0.222	p = 0.041*	p = 0.063	p = 0.082
Phosphate [mmol/L]	p = 0.347	p = 0.389	p = 0.209	p = 0.597
Magnesium [mmol/L]	p = 0.207	p = 0.858	p = 0.099	p = 0.316

In the last column total p value represents the difference between the control group and all the studied groups combined. Children with hypertension (B), overweight children with hypertension (C) and overweight children with normal blood pressure (D).

*BMI Percentile* body mass index percentile, *LDL cholesterol* low-density lipoprotein cholesterol, *HDL cholesterol* high-density lipoprotein cholesterol,* TG* triglycerides, *ApoA* apolipoprotein A1, *ApoB* apolipoprotein B.

*p < 0.05, **p < 0.01.

We have found statistically significant differences between all included patients and the control group in the values of vitamin D, BMI, systolic and diastolic pressure, homocysteine, HDL and LDL cholesterol, triglycerides, apolipoprotein B and uric acid.

Table 4 presents correlations between different variables and vitamin D in all the observed patients. Statistically significant correlations were found between vitamin D levels and BMI, systolic pressure, diastolic pressure, homocysteine, triglycerides, and apolipoprotein A1.

**Table 4:** Correlation between vitamin D levels and different parameters in all of the observed groups with the use of Pearson's correlation coefficient.

Variable	Vitamin D
BMI percentile	r = − 0.196, p = 0.010**
Systolic pressure [mmHg]	r = − 0.190, p = 0.002*
Diastolic pressure [mmHg]	r = − 0.149, p = 0.050*
Homocysteine [μmol/L]	r = − 0.208, p = 0.007**
Total cholesterol [mmol/L]	r = 0.040, p = 0.604
LDL cholesterol [mmol/L]	r = − 0.017, p = 0.832
HDL cholesterol [mmol/L]	r = 0.124, p = 0.115
TG [mmol/L]	r = − 0.196, p = 0.011*
Glucose [mmol/L]	r = − 0.090, p = 0.249
ApoA [g/L]	r = 0.222, p = 0.007**
ApoB [g/L]	r = − 0.042, p = 0.621
Alkaline phosphatase [µkat/L]	r = 0.096, p = 0.258
Uric acid [μmol/L]	r = − 0.156, p = 0.060
Calcium [mmol/L]	r = 0.050, p = 0.950
Phosphate [mmol/L]	r = − 0.104, p = 0.189
Magnesium [mmol/L]	r = − 0.027, p = 0.730

*BMI Percentile* body mass index percentile, *LDL cholesterol* low-density lipoprotein cholesterol, *HDL cholesterol* high-density lipoprotein cholesterol, *TG* triglycerides, *ApoA* apolipoprotein A1, *ApoB* apolipoprotein B.

*p < 0.05, **p < 0.01.

Model of multivariate analysis of variables presented in Table 5 confirms that BMI percentile, systolic pressure, diastolic pressure, homocysteine, triglycerides and apolipoprotein A1 all have statistically significant effect on vitamin D levels.

**Table 5:** Model of multivariate analysis of variables which proved significant in univariate analysis.

Variable	Standarized beta coefficient	Sig
BMI percentile	− 0.195	p = 0.012*
Systolic pressure	− 0.193	p = 0.010**
Diastolic pressure	− 0.230	p = 0.002**
Homocysteine	− 0.209	p = 0.001**
TG	− 0.197	p = 0.017*
ApoA	0.223	p = 0.007**

Vitamin D was used as the dependant variable.

*BMI percentile* body mass index percentile, *TG* triglycerides, *ApoA* apolipoprotein A1.

*p < 0.05, **p < 0.01.

Afterwards we checked the adjusted coefficient of determination: R^2^ = 0,462 which showed a moderate level of correlation. By using the multivariate analysis we concluded, that the variables in our model can explain 40.3% of the phenotype. The model was found to be statistically significant (F = 5.74, p < 0.001).

In Table 6 vitamin D levels in relation to different systolic blood pressure values are presented.

**Table 6:** Vitamin D values in comparison to systolic blood pressure percentiles.

	Systolic pressure under 85th percentile	Systolic pressure between 85 and 95th percentile	Systolic pressure above 95th percentile
Minimal value of vitamin D [nmol/l]	36.90	7.5	14.3
Maximal value of vitamin D [nmol/l]	148.50	126.8	125.40
Mean value of vitamin D [nmol/l]	70.91	63.49	59.44
Standard deviation (SD)	± 26.05	± 25.3	± 23.43

Hypertension is defined as at or above the 95th percentile.

The higher the systolic pressure, the lower the average levels of vitamin D are. There is statistically significant difference between the group with systolic blood pressure pressure below the 85th percentile and the group with systolic pressure above the 95th percentile (p = 0.01). There was no statistically significant difference between the group with systolic blood pressure below the 85th percentile and the group with systolic pressure ranging from the 85th to 95th percentile (p = 0.182) as well as between the latter and the group with systolic blood pressure over the 95th percentile (p = 0.398).

## Discussion

The purpose of this study was to find potential differences in vitamin D in groups of children with hypertension, overweight children, overweight children with hypertension and healthy control group as well as some correlations between vitamin D levels and different parameters. We discovered statistically significant differences in vitamin D levels in all groups in relation to the control group. There were also statistically significant differences between groups of overweight children and children with hypertension, as well as between children with hypertension and overweight children with hypertension. There were no significant differences in vitamin D levels between the groups of overweight children (group C and D). Our findings suggest there is a link between obesity and arterial hypertension, as well as vitamin D, which has also been proven by some other studies^10,17,18^. Considering the statistically significant differences between patients with hypertension and both groups of overweight patients (group C and D), we conclude that obesity presents a bigger negative predictive factor for vitamin D values than hypertension. The effects of both factors combined were not proven to affect vitamin D values significantly more than each individual factor. Significantly low levels of vitamin D in children with hypertension and normal BMI suggest vitamin D affects blood pressure independently of obesity. Similar observations have been made in some other studies^10,17–19^.

We discovered statistically significant correlations between vitamin D and various parameters in our research sample of 175 children. The correlation between BMI, measured in percentiles, and vitamin D was statistically significant, which supports our previous findings when comparing individual groups of children. The lower levels of vitamin D in overweight children are probably the result of volume dilution as well as reduced sunlight exposure. Vitamin D being fat-soluble also sequesters in body fat depots leading to lower bioavailability in the obese children^6,20,21^. When supplementing vitamin D in overweight children, in order to achieve appropriate vitamin D serum levels, higher doses are usually administered compared to the children, not overweight^6,18^.

Another statistically important correlation, albeit smaller, was discovered between vitamin D and systolic blood pressure. The reason for that likely being the presence of VDR in endothelial and smooth muscle cells of blood vessels and cardiomyocytes. Studies in recent years have provided evidence of the role vitamin D in reducing high blood pressure. This is achieved through inhibition of the renin-angiotensin hormone system, modulation of the endothelial function and reduction of oxidative stress in blood vessels^22,23^.

Together with the fact that there is a statistically significant difference between the group of normal-weight children with hypertension and the control group, we can assume that vitamin D is a non-obesity-dependent negative predictive factor of arterial hypertension and that by supplementing it, we could potentially improve blood pressure. However, it is important to keep in mind that a healthy body weight is what helps maintaining sufficient levels of vitamin D in the first place^24^.

Another correlation, which proved significant, was discovered between vitamin D and homocysteine, which is again in line with findings of other studies. This is most likely the consequence of vitamin D and its regulation of cystathionine-β-synthase (CBS), an enzyme that converts homocysteine into cystathionine, which is further converted into cysteine. The net effect of this reaction is a reduction of total blood homocysteine. Because hyperhomocysteinemia can lead to endothelial dysfunction, vitamin D can be seen as a potential protecting agent in preventing premature cardiovascular diseases^25–27^.

We have noticed a statistically significant negative correlation between vitamin D and TG. Another negative correlation was found between vitamin D and LDL cholesterol and a positive one between vitamin D and HDL cholesterol, however, it was statistically insignificant. These results are in line with findings of other similar studies, which proved correlation between low serum vitamin D levels and blood lipid abnormalities in children^18,24^. Statistically significant non-obesity-dependent correlation was established between vitamin D and TG, indicating vitamin D is an independent negative predictive factor of hypertriglyceridemia. This is likely because vitamin D lowers fatty acid absorption in the intestine by increasing the absorption of calcium and higher calcium levels promote the hepatic conversion of cholesterol into bile. Vitamin D also reduces lipogenesis and promotes lipolysis through parathyroid hormone (PTH) inhibition, which additionally helps regulate blood lipid parameters^26–28^.

Next, there was a statistically significant positive correlation between apo A and vitamin D. Apo A is one of the main components of HDL cholesterol, its presence is therefore an indicator of cardiovascular health. In terms of the role of apo A as a marker of cardiovascular health, it is important to consider its relationship with apo B because higher values of the latter indicate an increased risk for cardiovascular disease. In our study, we discovered a negative correlation between vitamin D and apo B, though insignificant. These results reflect the role of vitamin D in regulating the hepatic synthesis of apo A through its action on the VDR receptor^28–30^.

Finally, the negative correlation between vitamin D and uric acid, which we found to be just slightly below the limit of statistical significance, is most likely the consequence of 1-alpha-hydroxylase inhibition, which is caused by higher levels of uric acid. 1-alpha-hydroxylase is an enzyme that converts 25(OH)D into its biologically active form 1,25(OH)_2_D and therefore promotes its actions^31,32^.

Unfortunately, PTH determination was not performed in our study, which is one of its disadvantages. Namely, PTH and vitamin D are two major regulators of mineral metabolism, forming a tightly controlled feedback cycle, with PTH being a major stimulator of vitamin D synthesis in the kidney and vitamin D exerting negative feedback on PTH secretion^33^. They play critical roles in the maintenance of calcium and phosphate homeostasis as well as the maintenance of bone health. In vitamin D deficiency, higher concentration of PTH is expected, however, not leading to clinical hyperparathyroidism in milder deviations^34^. Both hyperparathyroidism and vitamin D deficiency have been implicated in a variety of cardiovascular disorders including hypertension, exerting direct effects on the endothelium, heart, and other vascular structures^32^. Therefore, we are of opinion that vitamin D deficiency alone is an indication for vitamin D supplementation according to guidelines^35^, and obtaining PTH value, probably elevated because of feedback due to the hypovitaminosis, does not affect clinical decision. Anyway, it might be important in relation to possible different cut-off values of vitamin D deficiency of different groups of patients which has been already found for obese children and should be also determined in a prospective future study for hypertensive ones^36^.

Despite many positive and promising findings of our study, there are still some things, which could be improved. Ideally, the groups would be comprised of equal number of patients of the same sex and age. This would be very difficult to achieve in our situation, as we recruited our patients in an anterograde fashion. Another thing, which could provide us with more relevant results would be to include a larger number of participants. The study could be upgraded by simultaneous PTH determination to find out the cut-off values of vitamin D deficiency which might be different in different groups of risk children, to better define the need for treatment as well as the optimal vitamin D doses.

We can conclude with relative certainty that the results of our study are in line with the findings of other recent studies, whether regional or international, and that they provide enough evidence to the theory that maintaining a healthy body weight and appropriate vitamin D levels is of vital importance, especially in patients subjected to cardiovascular diseases.

## Conclusion

Our pilot study has shown statistically significant differences in vitamin D serum levels between groups of children who were overweight, normal weight with hypertension, overweight with hypertension and the control group. There was also statistically significant correlation between vitamin D and blood pressure, BMI, and some blood lipid parameters. Our findings suggest the need for further research into vitamin D and its role in protecting cardiovascular health, as well as its use in therapeutic and preventive care for children with hypertension and/or obesity.
